# Hypertrophy

**Published:** 1860-11

**Authors:** 


### HYPERTROPHY.

From hyper, above, and trophe to nourish, used always in the abnormal sense when in reference to size of tissues. Nutrition is always extravascular—(is it always or ever extracellular?) Might not decomposition and recomposition of the cells occur in the manner of crystals of salts, from an excess of the proper solvent being present, favoring transudation through the thin membraneous vessel wall?

If nutrition be extravascular, that is, effected without the circulation along the tracks of the capillaries, then it follows that each tissue must have a formative organ also without the circulation proper, endowed with the power to select and elect the particular constituents needed for each specific locality, modified of course by normal and abnormal conditions; which define the limit of purely natural development and hypertrophy. Else all exosmosed fluids must be alike solutions of their various soluble constant or adventitious salts:—and thus produce not organs, but mere consolidations of one general plasma of conglomerate crystals under the law of crystallization, leaving the water set free thereby to make its own course out of the system by percolation.

Enamel organ secretes the materials from the circulation and crystallization completes the structure. This secretion exhausts the organ to complete obliteration:—rendering it impossible ever after to exert its function in either a hyper or hypo degree. So we can have no hypertrophy of enamel in human teeth.

Cement organ properly secretes the calcareous and gelatinous matter, which product is formed by crystallization in calciferous cells, which in turn give birth to the true cemental cell, and may be properly subject to true hypertrophy, the cell being a modified bone cell, usually more regular in conformation and smaller than the average of that in bone.

The ivory organ is the pulp proper. Hypertrophy of this tissue seems to be an impossibility. But calcification of the pulp in part or entire is of too frequent occurrence to be doubted. To be sure that proper hypertrophy is out of the question so far as dentine is concerned, it is only necessary to consider for a moment the distribution of the vessels of supply to the pulp at all times, but especially after the cap of the enamel has been completed and the whole crown of pulp proper embraced in the calcified inner cap (dentine)

which cuts off the possibility of its receiving constituents from any other source than the ramifications of the artery of the future foramen at the apex of the fang.

The only approach to a claim to this term as applied to dentine is the "secondary dentine," or the true ossification of the pulp, which must be confined within the dentinal canal; with this rare modification i. e. where the dentinal tubes have been broken down for a part of the course of the length of their greater size, and replaced by this identical substance, "secondary dentine," in *this* instance a pathological product, whatever may be said of ordinary secondary dentine. In this matter the one to whom we owe the term, (Tomes) that actually accurate observer, has perpetrated or permitted a mistake, (of delineator or engraver) to pass uncorrected. I refer to the very regular and apparently original arrangement of the tubules in his 84th and 99th figures, which he claims as a clear case of secondary dentine interposing to preserve the life of the pulp. Now I only have this to say: the cut does not correctly represent the specimen or he is not warranted in denominating the barrier "secondary dentine." Does any ask why? Ans.—All secondary dentine is but poorly endowed with tubes and they are always in irregular, tortuous, sprangling tufts, or single wandering crooked lines amid an almost solid mass of calciferous substance—or so nearly approximating that form of dentine termed vaso-dentine that no one would mistake them for specimens of primal structures, if at all familiar with their microscopical appearances, the only method of becoming acquainted with the truth respecting these very fine structures.

Then let us have no more "reports" of the hypertrophy of dentine with or without *cuts*, until a new method of integration and disintegration shall have been inaugurated by the only competent authority—"*Madam Nature.*" K.

